# Linking Variability in Phytochemical Composition with Safety Profile of *Thymus carnosus* Boiss. Extracts: Effect of Major Compounds and Evaluation of Markers of Oxidative Stress and Cell Death

**DOI:** 10.3390/ijms25105343

**Published:** 2024-05-14

**Authors:** Carlos Martins-Gomes, Fernando M. Nunes, Amélia M. Silva

**Affiliations:** 1 Centre for Research and Technology of Agro-Environmental and Biological Sciences (CITAB), Cell Biology and Biochemistry Laboratory, University of Trás-os-Montes and Alto Douro (UTAD), Quinta de Prados, 5000-801 Vila Real, Portugal; camgomes@utad.pt; 2 Chemistry Research Centre-Vila Real (CQ-VR), Food and Wine Chemistry Laboratory, University of Trás-os-Montes and Alto Douro (UTAD), Quinta de Prados, 5000-801 Vila Real, Portugal; fnunes@utad.pt; 3 Department of Chemistry, School of Life Sciences and Environment, University of Trás-os-Montes and Alto Douro (UTAD), 5000-801 Vila Real, Portugal; 4 Department of Biology and Environment, School of Life Sciences and Environment, University of Trás-os-Montes and Alto Douro (UTAD), 5000-801 Vila Real, Portugal; 5 Institute for Innovation, Capacity Building and Sustainability of Agri-Food Production (Inov4gro), University of Trás-os-Montes and Alto Douro (UTAD), Quinta de Prados, 5000-801 Vila Real, Portugal

**Keywords:** cytotoxicity, reactive oxygen species, mitochondrial membrane potential, cell cycle arrest, apoptosis, necrosis, *Thymus carnosus* Boiss., phytochemicals

## Abstract

Natural products are generally considered safe for human consumption, but this classification is often based on ethnobotanical surveys or their use in traditional medicine over a long period of time. However, edaphoclimatic factors are known to produce different chemotypes, which may affect the safety profile and bioactivities, and are not commonly considered for plants exploited as crops worldwide. *Thymus carnosus* Boiss., a thyme species with various health-promoting effects, has potential pharmaceutical applications, but edaphoclimatic factors were found to significantly impact its phytochemical composition. Thus, we aimed to assess the safety profile of *T. carnosus* extracts obtained from plants harvested in two locations over three consecutive years and to establish an association with specific components, an essential study in the search for new sources of nutraceuticals. Thus, the antiproliferative effect of an aqueous decoction (AD), hydroethanolic (HE) extracts, and major extracts’ components of *T. carnosus* was evaluated on intestinal (Caco-2) and hepatic (HepG2) cell models, revealing effects dependent on extract type, cell line, and tested compounds. Flavonoids induced different cytotoxic patterns, which could be attributed to molecular structural differences. Flow cytometry analysis showed apoptosis and necrosis induction, mediated by the modulation of intracellular reactive oxygen species and mitochondrial membrane potential, effects that were dependent on the cell line and phytochemical composition and on the synergism between extracts components, rather than on the activity of an isolated compound. While ursolic acid was the component with the strongest impact on the difference between extraction methods, flavonoids assumed a pivotal role in the response of different cell lines to the extracts. We report for the first time, for *Thymus* spp. extracts, that variations in the phytochemical composition clearly influence the cellular response, thus highlighting the need for extract standardization for medicinal applications.

## 1. Introduction

*Thymus carnosus* Boiss. is a near-threatened species, endemic to the Iberian Peninsula. The endangered status is caused by anthropogenic activities, such as increased tourism and urbanization, which occur to the detriment of wild areas and natural reserves. This is the case for coastal areas and sand dunes, the habitat of *T. carnosus*. We have recently reported the potential of this species as a novel functional food and as a source of nutraceuticals. Both aqueous decoction (AD) and hydroethanolic (HE) extracts were identified as promising antitumoral, antidiabetic, neuroprotective, and antioxidant agents [1,2,3].

Currently, *T. carnosus* is not used for food or pharmaceutical applications, unlike other species in the *Thymus* genus that have also been shown to present various bioactivities, namely, *Thymus vulgaris* [4], *Thymus zygis* [5], *Thymus mastichina* [6], and *Thymus fragrantissimus* [7]. The main concern related to the globalized used of these natural resources as crops is the effect of the different edaphoclimatic conditions on their phytochemical composition, and how they may affect their bioactivities and safety profiles. To date, the effect of the variability in the phytochemical composition of thyme species extracts on their antiproliferative potential is still unclear. As a complex matrix comprising a wide range of bioactive compounds, it is mandatory that the relationship between phytochemical composition and cytotoxicity (or safety profiles) be assessed. In addition, it is crucial to investigate which extract molecules are the main influencers of toxic/safety effects. 

Regarding *Thymus* species, a study using extracts obtained from *Thymus longicaulis* C. Presl. leaves harvested in different time points between July 2012 and April 2013 analyzed the effect of harvest period on the extracts-induced antiproliferative activity in human cancer cell lines and a normal human lung fibroblast (MRC-5) cell line [8]. Significant variations were observed, between the different harvest times chosen, which also depended on the cell line. The concentration of the main compound identified in these extracts, rosmarinic acid (RA), ranged between ~13 µg/mL and ~3030 µg/mL. However, the extract presenting the highest RA content did not induce the highest cytotoxicity [8]. The extracts that induced the highest antiproliferative activity were found to be rich in flavonoid derivatives such as luteolin-*O*-(?)-hexoside and quercetin-*O*-(?)-hexoside [8].

Although the variation in the extracts’ safety profile induced by different harvest years or geographical location of *Thymus* species has been poorly addressed, this assessment was already performed for some plants of the Lamiaceae family. Al-Maharik et al., 2022 [9], analyzed the variation in *Rosmarinus officinalis* (currently known as *Salvia rosmarinus* Spenn.) essential oil composition in plants harvested in different locations and at varying altitudes (between 117 m and 1026 m), finding great variation in the main compounds, such as α-pinene (ranging between 13.07% and 51.36%) and 1,8-cineole (ranging between 4.81% and 37.82%) [9]. And, they showed that the antiproliferative activity in several cell line models was dependent on the harvest location and the cell model [9]. The aqueous and hydroethanolic extracts of *Lavandula pedunculata* (Mill.) Cav. plants originating from thirteen different locations produced significant variations in the antiproliferative activity of human hepatocarcinoma (HepG2), human breast adenocarcinoma (MCF-7), human lung carcinoma (NCI-H460), and human cervix adenocarcinoma (HeLa) cells [10].

Martins-Gomes et al., 2023 [3], described for the first time the variation in the phytochemical composition of *Thymus carnosus* extracts as being dependent on year and harvest location. In addition, Martins-Gomeset al., 2023 [3], reported a correlation between the variation in the phytochemical profile of extracts and the antioxidant, neuroprotective, and antidiabetic activities. The inter-annual stability of such activities provoked new interest on the use of this species in the human diet. It was shown that, despite the variation induced by the harvest year and location, all extracts retained significant neuroprotective activity (evaluated through the inhibition of acetylcholinesterase (between ~32% and 61%) and tyrosinase (between ~11% and 43%)) and antidiabetic activity (α-glucosidase inhibition between ~12% and 27%) [3]. Despite presenting the highest variation within the extracts analyzed, tyrosinase inhibition did not present a significant correlation with any individual component of the extracts. On the other hand, an orthogonal partial least squares-discriminant analysis (OPLS-DA) model allowed for the identification of eriodictyol, luteolin, and quercetin derivatives as the main contributors to acetylcholinesterase and α-glucosidase inhibitions. Despite the higher contribution of flavonoid derivatives, *T. carnosus* extracts presented a higher content in phenolic acids, such as salvianolic acids and RA [3].

A study on the antiproliferative activity of *T. mastichina* extracts reported that phenolic acids (~72% and ~74% HE and AD extract composition, respectively) had a significant impact in the extract’s cytotoxicity, as evaluated in human colorectal adenocarcinoma (Caco-2) cells exposed for 24 h to AD and HE extracts, showing IC_50_ values of ~71 µg/mL and ~221 µg/mL, respectively, and in HepG2 cells showing IC_50_ values of >500 µg/mL and ~265 µg/mL, respectively [6]. The extracts contained high quantities of salvianolic acids A isomer, B/E isomer, K, and I, which may have been highly correlated with the antiproliferative activity [6]. Additionally, a study focused on the composition of *T. carnosus* extracts, at two different vegetative stages, revealed high contents of salvianolic acid A isomer and salvianolic acid K (both extracts) [1,2]. AD extracts of *T. carnosus* [2] had a lower total flavonoid content than *T. mastichina* AD extracts [6], which could partly explain the reduced toxicity of *T. carnosus* AD extracts. Regarding HE extracts, *T. carnosus* extracts had high contents of the pentacyclic triterpenoids oleanolic and ursolic acids, while these were not identified in the *T. mastichina* extracts, which were reported to correlate with the significant antiproliferative activity observed for *T. carnosus* extracts [2,6].

Therefore, variations in the phytochemical profile may have implications for the safe use of these species if they are intended for inclusion in the human diet. It may also be of interest to select specific chemotypes with specific bioactivities and target them for different biomedical/nutraceutical applications, such as for antitumoral activity. Thus, in this study, we addressed whether an association between the safety profile of *T. carnosus* AD and HE extracts and the variation in the phytochemical composition could be established. Aiming at the inclusion of *T. carnosus* in the diet as a functional food or as a source of nutraceuticals, it is highly relevant to understand how its adaptation as a crop may affect its safe use for human consumption or potential as an antitumoral agent. For this, we analyzed the antiproliferative activity of AD and HE extracts of *T. carnosus* harvested in two different locations (L1: Arrábida Natural Park; L2: botanical garden of the University of Trás-os-Montes and Alto Douro) in three different years (2018, 2019, and 2020), and we further addressed the cellular mechanisms behind this bioactivity. In addition, as this species contains a wide variety of compounds present in other Lamiaceae species already used in the human diet in its phytochemical composition, it was expected that the data obtained in this study can be used to understand and predict the variations in its biological effects according to the phytochemical composition of the extracts obtained from other species. 

## 2. Results and Discussion

### 2.1. Safety Profile of T. carnosus Extracts and Their Main Phytochemicals

**Figure 1:**
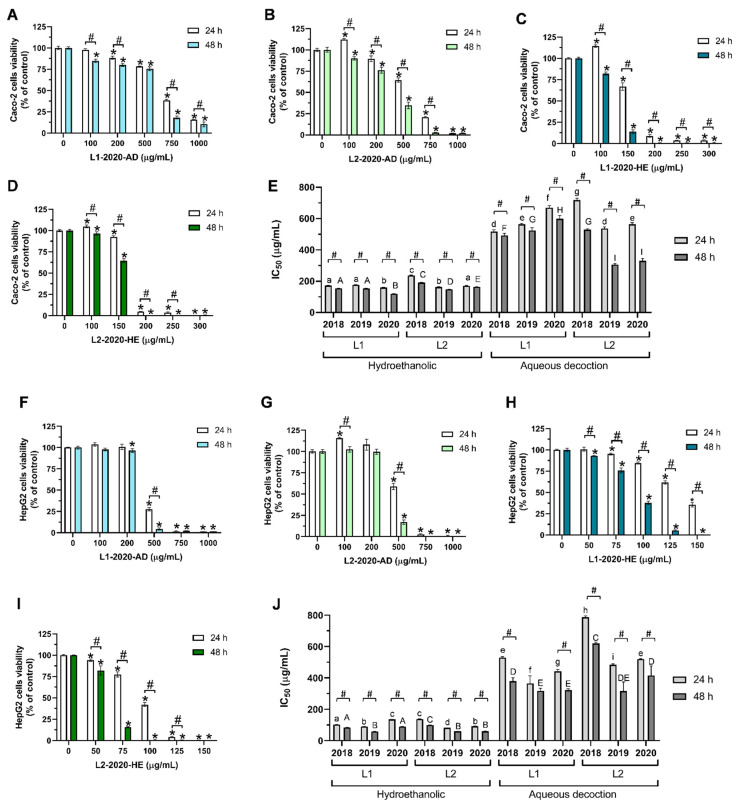
Safety profile of *T. carnosus* aqueous (AD) and hydroethanolic (HE) extracts (2020 harvest) evaluated in Caco-2 (**A**–**D**) and HepG2 (**F**–**I**) cells exposed for 24 h and 48 h to the extracts, as indicated. Significant statistical differences (when *p* < 0.05) between the control cells (nonexposed cells) and samples are denoted by “*” and between exposure times; those for the same concentration are denoted as “#” over a horizontal square bracket. IC_50_ values were calculated from three independent experiments (each one conducted in quadruplicate) for Caco-2 cells (**E**) and HepG2 (**J**) cells. Significant statistical differences (when *p* < 0.05) between the IC_50_ values are denoted with different letters (lowercase for 24 h, uppercase for 48 h exposure); those between exposure times for the same extract are denoted as “#” over a horizontal square bracket. Results are presented as mean ± SD.

The safety profile of *T. carnosus* AD and HE extracts was evaluated in Caco-2 and HepG2 cells using an Alamar Blue^®^ (Invitrogen; Alfagene, Lisboa, Portugal) assay. An initial screening was performed for all extracts at concentrations ranging between 100 and 1000 µg/mL. Due to the higher toxicity of the HE extracts, which was previously described for various extracts of *Thymus* species using the same extraction methods (e.g., *T. mastichina* [6] and *Thymus zygis* [5]), a different set of concentrations was used for the HE extracts (between 100 and 300 µg/mL for Caco-2 cells and 50 and 150 µg/mL for HepG2 cells). Figure 1 presents the data related to the cell viability assays in Caco-2 (panels A–E) and HepG2 cells (panels F–J) exposed to *T. carnosus* extracts, as indicated.

Overall, the extracts tested induced no or very low cytotoxicity (viability ≥ 95%) in Caco-2 and HepG2 cells at the lowest tested concentration (50 µg/mL or 100 µg/mL, depending on the extract tested) after a 24 h exposure (Figure 1 for the 2020 extracts; individual data used to calculate the IC_50_ values are not shown for 2018 and 2019 harvests). Dose- and time-dependent antiproliferative activity was observed for all extracts. From the datasets identical to those presented in Figure 1, the IC_50_ values for each extract were calculated for Caco-2 cells (Figure 1E) and HepG2 cells (Figure 1J) exposed to *T. carnosus* extracts from both harvest locations, performed in the three different years.

In Caco-2 cells exposed to the extracts for 24 h, L1-2020-HE produced the lowest IC_50_ (160.30 µg/mL), and the highest IC_50_ value was obtained for L2-2018-AD (718.60 µg/mL) (Figure 1E). A time-dependent effect was observed for all the extracts, as seen from the IC_50_ values. Overall, the HE extracts produced lower variation in the obtained IC_50_ values, ranging between 160.30 and 236.90 µg/mL, with L2-2018-HE being the outlier, as the IC_50_ values of the remaining extracts ranged between 160.3 and 177.90 µg/mL (Figure 1E).

These results correlate with the phytochemical composition reported by Martins-Gomes et al., 2023 [3], where L1-2020-HE had the highest phenolic and terpenoid concentrations, while L2-2018-HE had the lowest phytochemical concentration [3]. The AD-extracts-induced anti-proliferative activity resulted in IC_50_ values ranging between 517.80 and 718.60 µg/mL, i.e., higher IC_50_ values than those of HE, hypothetically due to a lower content of phenolic compounds and the absence of terpenoids (compared with HE). It should be noted that the extract that induced the highest anti-proliferative activity within the AD extracts, L1-2018-AD, was not the extract with the highest concentration in the phytochemicals identified and quantified [3]. However, the phytochemical composition (presented in [3]) once again directly correlated with the lower cytotoxicity of L2-2018-AD, which had the lowest phenolic compound content among all the extracts tested [3]. Notably, the Caco-2 cells exposed to the AD extracts obtained from the plant material harvested in L2 showed a more pronounced incubation-time-dependent decrease in cell viability, resulting in lower IC_50_ values after 48 h of exposure to the extracts when compared with that of the extracts from L1.

In the present study, we addressed the potential link between the variation in phenolics or terpenoids and the safety profile. Although phenolic compounds have been the most-addressed phytochemicals when considering the chemical composition of aromatic and medicinal plants and their correlations to potential bioactivities, in this work, we focused on the potential role of both phenolics and terpenoids. The phytochemical profile determined using HPLC-DAD-ESI-MS^n^ by Martins-Gomes et al., 2023 [3], was optimized to mainly analyze the presence of phenolics and terpenoids. However, other phytochemicals from different classes may have been present in these extracts but were not identified. In addition, other water-soluble compounds such as proteins or sugars may have contributed to the observed bioactivities. 

The IC_50_ values obtained for the HepG2 cells exposed to the *T. carnosus* extracts (Figure 1J), overall, were lower than those obtained for Caco-2 cells (Figure 1E). These results differ from those of other thyme species, such as *Thymus capitellatus* [11], *Thymus pulegioides* [12], or the antiproliferative activity of *T. carnosus* in these cell lines [1], where HepG2 cells were less sensitive to the extracts’ action. This difference may be due to a specific component present in the *T. carnosus* extracts used in the present study or to the synergism between compounds, which is addressed below. As seen for Caco-2 cells, L2-2018-AD was the extract with a higher IC_50_ value in HepG2 cells (786.00 µg/mL; 24 h exposure; Figure 1J), while the lowest IC_50_ value was obtained using L2-2019-HE (81.53 µg/mL; 24 h exposure), which did not present the highest phytochemical concentration. In fact, both HE extracts obtained from the plant material harvested in 2019 produced the highest anti-proliferative activity, regardless of the location. These results also corroborate the hypothesis that HepG2 cells are more sensitive to a specific component or to the synergy of various compounds within the extract. The IC_50_ values for the HE extracts ranged between 81.53 and 137.70 µg/mL, while for the AD extract, they ranged between 364.70 and 786 µg/mL in HepG2 cells exposed to the extract for 24 h. Unlike the HE extracts, the phytochemical composition directly correlated with the cytotoxicity observed for the AD extracts, as L1-2019-AD produced the lowest IC_50_ value, as it was the AD extract that presented the highest phenolic compound contents [3]. With the exception of the L1-2019-AD extract, a time-dependent effect was observed for the IC_50_ value of all extracts (Figure 1J).

According to the results obtained, the year 2020 was chosen for assays related to the cell death mechanisms, as it produced the extract with the highest contents of most components: the HE extract of plant material harvested at L1 (Arrábida Natural Park). Concerning the phenolic compounds in the HE extracts, as seen in Table 1, RA was the major component, being present at a concentration higher than that of the sum of the salvianolic acids or the luteolin derivatives. In the AD extracts, the sum of salvianolic acids represented the major fraction in L1-2020-AD, and luteolin derivatives were the major fraction in L2-2020-AD (Table 1). As described above, L1-HE was the extract with the highest RA, total salvianolic acid, total luteolin derivative, and total quercetin derivative contents. The pentacyclic triterpenoids oleanolic (OA) and ursolic (UA) acids were the major compounds in the HE extracts but were not being present in the AD extracts, which may thus be the determinants of the antiproliferative activities [2]. In addition, the 2020 harvest produced a different pattern when comparing the IC_50_ values between the cell lines. For the HE extracts, L1-2020-HE produced an IC_50_ value for Caco-2 cells that was ~1.18 times higher than that for HepG2 cells; the IC_50_ value produced by L2-2020-HE was 1.86 times higher. While L2-2020-AD produced an IC_50_ value in Caco-2 cells that ~1.08 times higher than that in HepG2 cells, the IC_50_ value produced by L1-2020-AD in Caco-2 cells was 1.52 times higher than that in HepG2 cells (Figure 1). This variation therefore depended on the cell line used and should be further addressed to understand the safety profile and the potential bioactivities targeting different tissues.

Aiming to determine the individual role of the main compounds present in the *T. carnosus* extracts regarding the antiproliferative activity shown in Figure 1, the cytotoxic effect of the individual components was also a parameter that was analyzed. However, due to the complexity of this matrix and the lack of commercial standards available for all the identified components, only the major compounds were considered in this study. When a commercial standard was not available, aglycone was used. More precisely, the effects of RA, salvianolic acid A (SAA), luteolin-7-*O*-glucoside (L-7-G), eriodictyol, quercetin, OA, and UA were studied. For a better understanding, a summary of the main components of the *T. carnosus* AD and HE extracts from the plant material harvested in 2020 is presented in Table 1, as well as a sum of the compounds with structural similarities (e.g., salvianolic acid or luteolin derivatives), and the concentrations are presented in µM.

**Figure 2:**
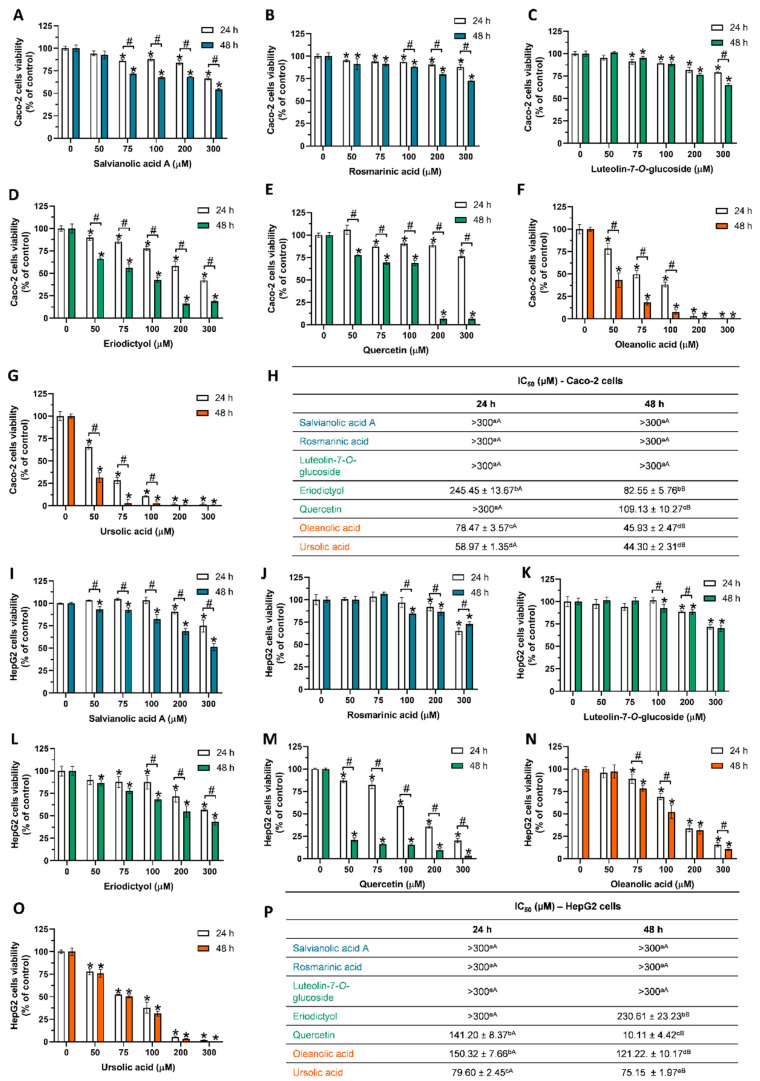
Antiproliferative activity of the main phytochemicals identified in *T. carnosus* AD and HE extracts evaluated in Caco-2 (**A**–**G**) and HepG2 (**I**–**O**) cells exposed for 24 h and 48 h to the extracts. The IC_50_ values were calculated from three independent experiments (each one conducted in quadruplicate) for Caco-2 cells (**H**) and HepG2 cells (**P**). Significant statistical differences (when *p* < 0.05) between the control cells (nonexposed cells) and samples are denoted by an “*”; those between exposure times for the same concentration are denoted as “#” over a horizontal square bracket. For IC_50_ values, significant statistical differences are denoted with different lowercase letters between phytochemicals at the same exposure time and with different uppercase letters and between exposure times for the same phytochemical. Results are presented as mean ± SD. In panels (**H**,**P**), different classes of compounds are denoted by different colors, as follows: phenolic acids (blue), flavonoids (green) and pentacyclic triterpenoids (orange).

Figure 2 presents the antiproliferative effect of the individual phytochemicals and the respective IC_50_ values obtained for Caco-2 and HepG2 cells. Regarding the phenolic acids, SAA induced higher cytotoxicity than RA, both in Caco-2 and HepG2 cells, although it did not reduce cell viability below 50% at concentrations up to 300 µM (Figure 2H,P). In Caco-2 cells exposed to 300 µM SAA for 48 h (Figure 2A), we observed a cell viability of 54.40%, while RA reduced cell viability to 72.56% under the same conditions (Figure 2B). Comparing the cell lines, SAA induced lower cytotoxicity in HepG2 cells at 24 h and 48 h exposure than in Caco-2 cells (Figure 2A,I). Regarding RA, when exposed to 300 μM of this phenolic acid for 24 h, HepG2 cells presented lower viability (65.22%) than Caco-2 cells (87.76%), but, once again, there were no major differences at 48 h of exposure (Figure 2B,J).

Contrasting the identical patterns of antiproliferative activity induced by phenolic acids, the tested flavonoids induced different cytotoxic patterns, which were attributed to the differences in the molecular structures. Like RA and SAA, L-7-G did not reduce cell viability below 50% at concentrations up to 300 µM (Figure 2H,P). L-7-G reduced Caco-2 cell viability at concentrations ≥75 µM (*p* < 0.05), but for HepG2 cells, a significant effect was only observed at concentrations ≥ 100 µM (48 h exposure). L-7-G, at the highest tested concentration (300 µM; 48 h,) produced reductions of ~35% and ~30% in Caco-2 and HepG2 cell viability, respectively (Figure 2C,K). Eriodictyol induced higher antiproliferative activity than L-7-G in both cell lines, with the effect also being dependent on the cell line (Figure 2D,M). In HepG2 cells, eriodictyol reduced cell viability to 56.87% and 43.18%, at exposures of 24 h and 48 h, respectively; in Caco-2 cells, the decrease in cell viability was more pronounced, and exposure to eriodictyol significantly reduced cell viability at all tested concentrations (Figure 2D). And, after 48 h of exposure, eriodictyol reduced Caco-2 cell viability by ~81% (cell viability ~19% of control), resulting in an IC_50_ of 82.55 µM (Figure 2H).

On the other hand, quercetin-induced cytotoxicity presented an opposing pattern, as HepG2 cells were found to be more sensitive to this flavonoid. For Caco-2 cells exposed to quercetin for 24 h only at concentrations ≥75 µM, a significant decrease in cell viability was observed (Figure 2E); in HepG2 cells, all quercetin concentrations tested produced significant cytotoxicity (Figure 2M). At 48 h of exposure, the cell-line-dependent effect was notable at concentrations between 50 and 100 µM, with Caco-2 cell viability being ≥68%, while HepG2 cell viability, at these concentrations, was ≤20%. This resulted in the significantly lower IC_50_ values for the HepG2 cells in Figure 2P of 141.20 µM (24 h) and 10.11 µM (48 h), contrasting the IC_50_ values for Caco-2 cells exposed to quercetin for 48 h (109.13 µM; Figure 2H). These results demonstrated that among the tested flavonoids, the impacts of eriodictyol and quercetin on the antiproliferative profile of *T. carnosus* extracts were probably more prominent than that of L-7-G. In addition, quercetin was expected to play a significant role in the higher cytotoxicity induced by the *T. carnosus* extracts in HepG2 cells (Figure 1). 

As mentioned above, we expected the pentacyclic triterpenoids oleanolic and ursolic acids to justify, in part, the higher toxicity observed for the HE extracts. As shown in Figure 2F (OA) and 2G (UA), among the tested phytochemicals, the terpenoids induced the highest cytotoxicity in Caco-2 cells, with a significant cell viability reduction at the lowest tested concentration (50 µM) at both exposure times. Comparing the two compounds for a 24 h exposure, ursolic acid induced higher cytotoxicity than oleanolic acid, resulting in an IC_50_ value 1.33 times lower; however, the IC_50_ values obtained at 48 h of exposure of Caco-2 cells to the two terpenoids were identical (*p* > 0.05). 

Due to their poor water solubility and high lipophilicity [13,14], both OA and UA may have higher affinity to cell membranes, which is supported by their high partition coefficient [15]. In fact, other authors reported that OA and UA can modulate the fusion, fluidity, and permeability of biological membranes [16], which may have been related to the increased cytotoxicity observed in the Caco-2 cells exposed to pentacyclic triterpenoids. Comparing both cell lines, HepG2 cells (Figure 2N,O) presented higher resistance to the action of oleanolic and ursolic acids, having higher IC_50_ values than Caco-2 cells under all tested conditions. Contrary to what was observed for Caco-2 cells, among the phytochemicals tested, terpenoids did not produce the highest toxicity in HepG2 cells, given that the IC_50_ (at 48 h) values of quercetin were 11.99 and 7.43 times lower than the IC_50_ values of oleanolic and ursolic acids, respectively (Figure 2P).

Comparing the results here obtained with those in the literature, a study that described the effect of RA on Caco-2 cells indicated that concentrations ≤ 90 µM did not reduce cell viability [17]. In HepG2 cells, Ma et al., 2018 [18], reported a significant reduction in cell viability induced by RA at concentrations ≥34.69 µM, with an IC_50_ (48 h) of 91 µM, a value lower than that here reported (>300 µM; Figure 2P). A different study reported that no significant cytotoxicity was observed in HepG2 cells exposed for 24 h or 48 h to RA at concentrations up to 160 µM [19]. Regarding the antiproliferative activity of SAA on Caco-2 cell viability, to the best of our knowledge, this is the first study reporting a time- and dose-dependent cytotoxicity in this cell line. Notably, a single study addressed the antitumoral potential of SAA in in vitro colorectal cancer models (DLD-1, Dukes-type C colorectal adenocarcinoma, and HCT-116 human colorectal carcinoma cells), reporting that SAA inhibited the secretion of glucose-regulated protein 78 (GRP78), a protein that promotes tumor proliferation, drug resistance, and angiogenesis [20]. However, no or low cytotoxicity was observed at concentrations up to 50 µM [20], which is in line with the results here presented (Figure 2A). In HepG2 cells, Li et al., 2020 [21], reported a cell viability of ≥ 80% for cells exposed to SAA at concentrations up to 250 µM, which is also in line with the results here presented (Figure 2I). The antiproliferative activity of SAA was evaluated in other cell lines, such as HeLa cells, human lung adenocarcinoma (H1975) cells, human prostate carcinoma (DU 145) cells, and human lung cancer (A549) cells, all presenting IC_50_ values ≤ 30 µM [22]. In two nasopharyngeal carcinoma cell lines (HONE-2 and NPC-39) exposed to 50 µM SAA, no reduction in cell viability was observed [23]. Salvianolic acid B, whose isomer was found in *T. carnosus* extracts [3], was also reported to induce antiproliferative activity in colorectal cancer, HCT-8 [24], HTC-116, HT-29 [25], and HepG2 cells [26]. 

Regarding the flavonoids tested, one study indicated that exposing Caco-2 cells to L-7-G, for 48 h, significantly reduces cell viability to 50% of control, at ~55 µM [27], a value much lower than the one here presented (Figure 2C). Another study reported a 38% reduction in the viability of Caco-2 cells exposed for 24 h to 223 µM L-7-G [28], a value similar to that presented in Figure 2C. L-7-G was shown to reduce HepG2 cells’ viability to ~50% at 44.60 µM [29], a value also lower than the one here reported (Figure 2K).

The antiproliferative effect of eriodictyol in human cell models has been less described in the literature, with cytotoxicity induced by flavonoid being reported at concentrations ≥ 5 µM (24 h) in normal and tumoral human nasopharyngeal cells [30], an antiproliferative activity more prominent than that reported in this study (Figure 2D,L). 

Regarding quercetin, Min and Ebeler, 2009 [31], reported that Caco-2 cells exposed to 100 µM quercetin for 24 h showed no changes in cell viability, a result comparable to those shown in Figure 2E, where a cell viability of ~90% for concentrations up to 200 µM can be observed. Another study addressed the effect of quercetin and its glycoside derivatives on Caco-2 cell proliferation [32], showing that cells exposed to 50 µM quercetin for 48 h had a viability of approximately 75–80% [32], which is in line with the cell viability of 77.64% for 50 µM quercetin shown in Figure 2E. Of interest, given the content of several quercetin glycoside derivatives in *T. carnosus* extracts [3], according to Delgado et al., 2014 [32], the glycosidic derivatives of quercetin (quercetin-3-*O*-glucoside and quercetin-3-*O*-glucuronide) produced lower cytotoxicity in Caco-2 cells than aglycone [32]. In HepG2 cells, quercetin was found to reduce cell viability at concentrations ≥33 µM (24 h of exposure) [33], and, in the present study (Figure 2M), quercetin also reduced cell viability at concentrations ≥50 µM (the lowest concentration tested), and an IC_50_ (24 h) of 141.20 µM was obtained (Figure 2P).

The antiproliferative effect of the pentacyclic triterpenoids tested here, OA and UA, has also been addressed in previous studies. OA was found to reduce Caco-2 and HepG2 cell viability at concentrations ≥5 µM (24 h) and ≥2 µM (48 h), respectively [34,35]. However, another study reported an IC_50_ > 100 µM for HepG2 cells exposed to OA for 72 h, and an IC_50_ = 41 µM for UA [36], with these values being identical to those here presented (Figure 2P).

Therefore, aiming to understand the effect and individual contribution of different phytochemicals to the antiproliferative activity, as well as the impact of this effect on the safety profile of the total extract, several tests were carried out in order to quantify the biomarkers related to proliferation and/or cell death. These assays were also intended to better understand the mechanisms underlying the activity of the extracts and their individual compounds.

### 2.2. Effect of T. carnosus Extracts and Their Main Phytochemicals on Oxidative Stress and Mitochondrial Membrane Potential of Caco-2 and HepG2 Cells

**Figure 3:**
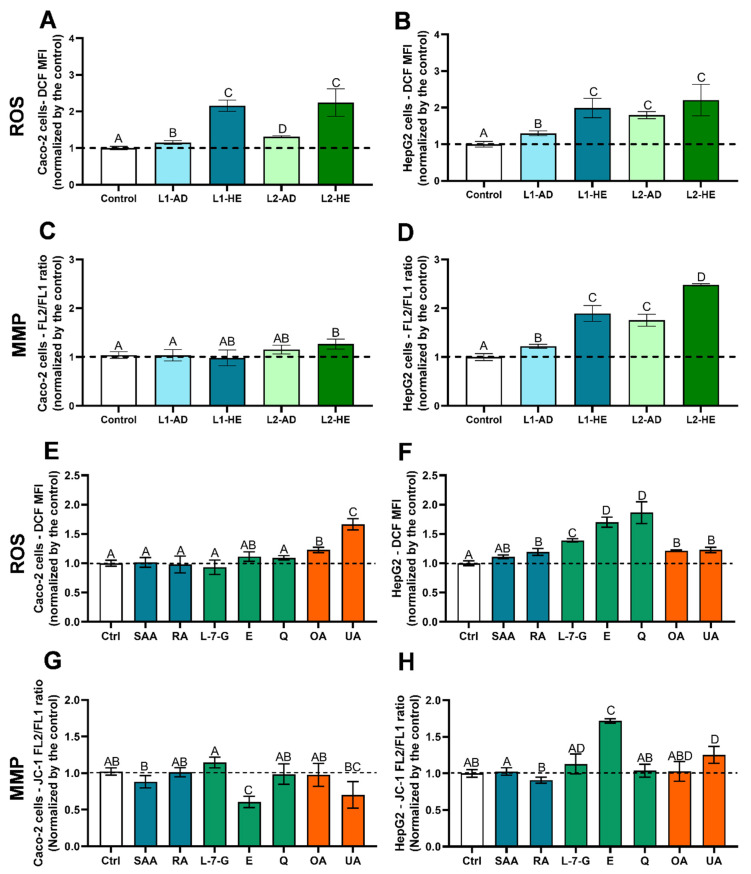
Intracellular reactive oxygen species (evaluated as DFC MFI; panels (**A**,**B**,**E**,**F**)) and mitochondrial membrane potential (MMP) modulation (evaluated as JC-1 fluorescence (FL2/FL1) ratio; panels (**C**,**D**,**G**,**H**)) induced by *T. carnosus* extracts (2020 harvest) at 100 µg/mL and by their main components (at 50 µM) in Caco-2 and HepG2 cells (24 h of exposure), as indicate. MFI: mean fluorescence intensity. Significant statistical differences (when *p* < 0.05) between samples are denoted with different letters. Results are presented as mean ± SD.

With the aim of investigating the mechanisms underlying the previously observed antiproliferative (cytotoxic) effect, we studied the effect of the extracts and their individual compounds on modulating the level of intracellular reactive oxygen species (ROS), the mitochondrial membrane potential (MMP), and the induction of apoptosis vs. necrosis. As previously mentioned, the extracts obtained from the plants harvested in 2020 were used in these tests, as well as the main compounds already indicated in Section 2.1. The results obtained for intracellular ROS and MMP modulation are presented in Figure 3.

For the assays related to the assessment of the intracellular levels of ROS, MMP, and apoptosis/necrosis by flow cytometry, a concentration of 100 µM was used for *T. carnosus* AD and HE extracts and 50 µM for the individual compounds. The concentration chosen for the extracts was based on the lowest IC_50_ obtained for extracts from the 2020 harvest (cells exposed for 24 h), which was 92.25 µM (L2-2020-HE; Figure 1J). Regarding the isolated phytochemicals, the lowest concentration tested in the cell viability assays (50 µM) was considered, as it showed no/low cytotoxicity for most phytochemicals and was higher than the concentration of each compound in the extracts, as presented in Table 1.

Regarding extract-induced oxidative stress, the results show a clear distinction dependent on the extraction method (AD vs. HE) and the cell line used. Despite not being cytotoxic to Caco-2 cells at 100 µM, when evaluated with an Alamar Blue assay, all extracts induced a significant (*p* < 0.05) increase in intracellular Caco-2 cell ROS levels, evaluated as dichlorofluorescein mean fluorescence intensity (DCF MFI; Figure 3A). However, while the AD extracts from L1-2020 and L2-2020 induced increases of 1.15 and 1.31 times (compared to the negative control, i.e., nonexposed cells), respectively, L1-2020-HE and L2-2020-HE induced 2.16- and 2.24-fold increases, respectively, data that corroborate the higher toxicity of the HE extracts. In the HepG2 cells (Figure 3B), it was also observed that all extracts significantly increased the intracellular ROS levels. While the HE extracts induced similar ROS levels in both cell lines, the AD extracts induced a greater effect on the oxidative stress in the HepG2 cells, showing increases of 1.30- and 1.79-fold for L1-2020-AD and L2-2020-AD, respectively, which may have contributed to the reduced cell viability observed in HepG2 cells (Figure 1). To the best of our knowledge, this is the first study reporting the effect of *T. carnosus* extracts on oxidative stress evaluated either in in vitro or in vivo experimental models. 

Comparing the extracts from different *Thymus* spp., N. Adham et al., 2020 [37] studied the effect of a chloroform fraction obtained from the methanolic extracts of *T. vulgaris* on human plasmacytoma myeloma cells (NCI-H929) after 1 h of exposure to concentrations between 3.20 µg/mL (~four-fold increase in ROS) and 25.90 µg/mL (~eight-fold increase in ROS) [37]. Thus, this *T. vulgaris* extract induced a significantly higher increase in intracellular ROS, with a shorter exposure time, than the *T. carnosus* extracts presented in this study (Figure 3); this emphasizes that the effect is dependent on the extraction method, extract composition, and cell line used.

Figure 3E (Caco-2 cells) and 3F (HepG2 cells) present the results of the phytochemicals-induced changes in the intracellular ROS. In Caco-2 cells, the pentacyclic triterpenoids were the only phytochemicals inducing an increase in ROS, which is in agreement with the higher cytotoxicity observed at 50 µM (Figure 2) when compared with the other phytochemicals tested. Furthermore, the ROS increases induced by OA and UA were significantly higher in Caco-2 cells than in HepG2 cells, which is also in line with the higher toxicity observed in Caco-2 cells (Figure 2F,G) than in HepG2 cells (Figure 2L,O) at the same time point. This also explains the different ROS levels produced by the AD extracts (which did not contain OA or UA) and HE extracts (rich in OA and UA).

Also in line with the results described above is the higher cytotoxicity observed for the HepG2 cells exposed to quercetin, as this flavonoid induced a significant increase in ROS, being the main contributor for this effect in HepG2 cells but not in Caco-2 cells. Indeed, as shown in Figure 2, quercetin induced the highest toxicity in HepG2 cells, with the lowest IC_50_ obtained in this study (Figure 2P). In addition, eriodictyol, L-7-G, RA, OA, and UA also produced a significant (*p* < 0.05) increase in intracellular HepG2 ROS levels. Therefore, all tested compounds, with exception of SAA, contributed to the *T. carnosus* HE-extract-induced oxidative stress in HepG2 cells, while for AD extracts, we observed flavonoids-induced oxidative stress. Nevertheless, analyzing Table 1, it can be observed that L1-2020-AD had a higher flavonoid derivative content than L2-AD-2020 but had a significantly lower ROS content (Figure 3B), implying that a synergism between the different antioxidant/pro-oxidant activities of the various compounds modulated the general redox state of the cells, giving a different result from that observed for each individual compound. The role of the phytochemicals present in *Thymus* spp. extracts in the modulation of both the response to oxidative damage as well as the induction of oxidative damage was recently reviewed [38].

Concerning the modulation of MMP, once again, a cell-line-dependent effect was observed; as in HepG2 cells, all extracts induced mitochondrial membrane hyperpolarization, with a higher MMP than in nonexposed cells (Figure 3D). In Caco-2 cells, only L2-2020-HE induced a slight increase in MMP (Figure 3C). In HepG2 cells, the increase in MMP followed the same pattern as observed for the ROS increase, which was expected since the mitochondrial membrane potential and thus mitochondria integrity are dependent on the intracellular ROS level [39]. The number of studies reporting the effect of thyme extracts on MMP modulation is low. A study using the *T. vulgaris* extract mentioned above induced an increase in ROS levels in NCI-H929 cells and modulated MMP in concentrations between 3.20 and 25.90 µg/mL, with a loss of MMP being observed, which was also analyzed using the JC-1 probe [37]. *T. vulgaris* essential oil also induced mitochondrial membrane depolarization in MDA-MB-231 cells at concentrations between 50 and 100 µg/mL [40]. The involvement of the dysregulation of the mitochondria potential in cell death has been widely described, mainly through the relationship between the loss of MMP and the release of proapoptotic factors into the cytoplasm such as cytochrome c, apoptosis-inducing factor (AIF), and second mitochondria-derived activator of caspases (SMAC), with the further activation of caspases, leading to apoptosis [41]. However, it is also known that mitochondrial membrane hyperpolarization may occur during the apoptotic process, also leading to cytochrome c release. This process may be transient and is possibly related to the closure of the voltage-dependent anion channels present in the outer mitochondrial membrane, which impairs ADP/ATP transport to/from the cytoplasm, while the exchange still occurs in the inner membrane [41,42,43]. This leads to membrane hyperpolarization and the generation of an osmotic gradient, causing the swelling of the mitochondrial matrix, which may lead to rupture. Upon membrane rupture and proapoptotic factor release, the loss of MMP is observed [41,42,43].

Analyzing the effect of individual phytochemicals on MMP variation, it was observed that in Caco-2 cells, there was a significant loss of MMP when cells were exposed to 50 µM of SAA, UA, and, most notably, eriodictyol (Figure 3G). In the HepG2 cells, RA induced a slight decrease in MMP, and UA induced a slight increase, while eriodictyol produced the greatest variation in MMP. But, unlike the effect on Caco-2 cells, eriodictyol induced the hyperpolarization of the mitochondrial membrane of HepG2 cells, similar to what was observed for the *T. carnosus* extract, thus leading to the hypothesis that this was the component with the largest contribution to the effect of the extract. However, the extract with a higher eriodictyol derivative content (L1-2020-AD) induced a lesser increase in MMP, probably due to a synergism with RA or another compound. Given the complexity of the matrix under study, synergistic effects between all components must be considered, and future studies should address the effect of the glycosylation of compounds on MMP modulation, as the effect of glycosylated derivatives may differ from that of the respective aglycone. Also, as mentioned above, the hyperpolarization of the mitochondrial membrane is assumed to be transitory, followed by a loss of MMP. Therefore, future studies should be performed to evaluate these parameters over time to assess the evolution of MMP during the cell death process and at several time points of exposure to extracts or individual phytochemicals. 

Of the phytochemicals tested in the present study, some were also described as modulators of MMP in other studies. L-7-G (20, 40, and 80 µM) was shown to induce MMP depolarization, cell cycle arrest in the S and G2/M phases, as well as apoptosis in nasopharyngeal carcinoma cells (NPC-039 and NPC-BM) [44]. SAA (50 and 100 µM) was also shown to induce the loss of MMP in human small cell lung cancer cells (SCLCs), accompanied by DNA damage and the induction of apoptosis [45]. The SAA-mediated loss of MMP was also observed here in Caco-2 cells (Figure 3G).

Quercetin was shown to induce the loss of MMP and apoptosis in murine melanoma cells (B16-BL6 cells) [46]. In human prostate cancer cells, quercetin (40 µM) also induced the loss of MMP, increased ROS, and induced apoptosis [39]. Eriodictyol was shown to reduce the MMP in A549 cells [47] at concentrations ≥25 µM, similar to the results obtained here in Caco-2 cells exposed to 50 µM eriodictyol (Figure 3G). Zhang et al., 2020 [47], also reported cell cycle arrest in the G2/M phase and mitochondrial-mediated apoptosis in A549 cells exposed to eriodictyol.

However, in studies with other phytochemicals, it was found that curcumin also induces mitochondrial membrane hyperpolarization in HepG2 cells, followed by depolarization, at concentrations between 27 and 108 µM, which led to cytochrome c release, thus triggering apoptosis [48]. The transient increase in MMP was associated with increased oxygen consumption and mitochondrial respiration [48], which is known to be positively correlated with increased ROS and is probably what happened in the hepatocyte cell model (HepG2 cells) due to its action in metabolizing xenobiotics [49]. Regarding pentacyclic triterpenoids, both OA and UA were shown to reduce MMP at concentrations ranging between 20 and 80 µM in hepatocarcinoma cells (HuH7) [50]. However, a study in human glioblastoma cells (DBTRG-05MG) reported that UA may also induce a transient hyperpolarization of the mitochondria membrane [51].

### 2.3. Evaluation of Cell Cycle Arrest Induced by T. carnosus Extracts and Their Main Phytochemicals

**Figure 4:**
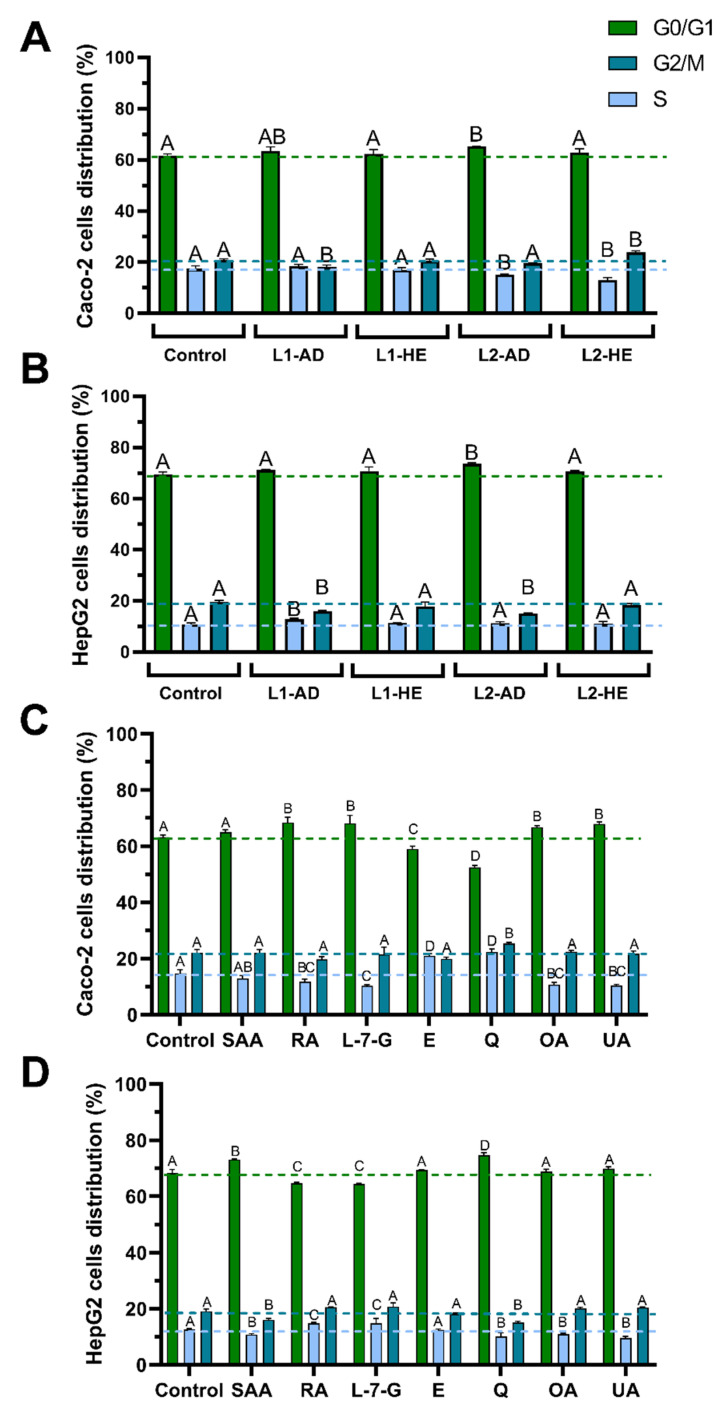
Cell cycle arrest induced by *T. carnosus* extracts ((**A**,**B**); 100 µg/mL; 2020 harvest) and their main components ((**C**,**D**); 50 µM) in Caco-2 and HepG2 cells (24 h of exposure). Significant statistical differences (when *p* < 0.05) between samples are denoted with different letters. Results are presented as mean ± SD. To better visualize and compare the effect of extracts/compounds on cell cycle arrest, a dashed line was placed defining the average value of each population in control (untreated) cells.

As discussed above, in studies concerning natural products that induce an increase in ROS and modulate MMP, effects on cell cycle arrest and apoptosis induction are also reported. For this reason, the effect of *T. carnosus* extracts (studied in this work), and its main phytochemicals, on the progression of the cell-cycle of Caco-2 and HepG2 cells was also addressed, as well as the relation of these results with the observed cytotoxicity. Results are presented in Figure 4.

Of the extracts tested, only L2-2020-AD induced the same effect in Caco-2 (Figure 4A) and HepG2 cells (Figure 4B), which was a cell cycle arrest in G0/G1, as observed by the increased percentage of cells in this phase. Of all the extracts tested, L2-2020-AD had the lowest content of the various phytochemicals, as summarized in Table 1; however, it induced a significant increase in ROS and MMP modulation when compared to L1-2020-AD (Figure 3). This further supports the idea that the synergistic effect of and the interaction between the phytochemicals present in the extracts may be more significant than the effect of the concentration of each individual compound. Concerning the HE extracts, L2-2020-HE induced cell cycle arrest in Caco-2 cells, with an increase in the G2/M population and a reduction in the percentage of cells in the S phase (Figure 4). A previous study using Caco-2 cells exposed to *T. carnosus* extracts reported that AD extracts induced cell cycle arrest in the S and G2/M phases, while HE extracts arrested cells in the G0/G1 phase [1]. This effect was thought to be mediated by high contents of RA and SAA, with the additional effects of the OA and UA in the HE extracts [1]. In fact, analyzing the results (Figure 4C), RA, L-7-G, OA, and UA induced cell cycle arrest in G0/G1 in Caco-2 cells.

The major reason accounting for this difference is that the extract used by Martins-Gomes et al., 2019 [1] (whose chemical characterization was published in [2]), was obtained from plant material harvested in a different vegetative stage, which thus had higher contents of phenolic acids and ursolic acid but a lower content of flavonoids. Significant cytostatic activity was observed for quercetin, which, in Caco-2 cells, induced significant cell cycle arrest in the S and G2/M phases, but, in HepG2 cells, the cell cycle arrest was observed in the G0/G1 phase. Likewise, rosmarinic acid and L-7-G induced cell cycle arrest in both cell lines but in different phases, depending on the cell line (Figure 4). Highly relevant is the fact that UA induced cell cycle arrest (G0/G1) in Caco-2 cells, in which increases in ROS and the loss of MMP were also observed (Figure 3E,G).

On the other hand, no UA-induced cytostatic effect was observed in HepG2 cells; however, the ROS increase was less noticeable, and the hyperpolarization of the mitochondrial membrane was observed (Figure 3F,H). This led to the hypothesis that different cell death processes were induced in the two cell lines.

RA-induced cell cycle arrest in G0/G1 was found in colorectal cancer cell lines (CT26 and HCT-116) [52]. Cell cycle arrest induced by SAA in the G0/G1 or G2/M phases in murine embryonic fibroblasts (3T6) was found to be dose-dependent; UA also induced a cytostatic effect in the G0/G1 phase in colorectal cancer (HCT15) cells [53]. Regarding the flavonoids, L-7-G was found to induce cell cycle arrest in the S and G2/M phases in nasopharyngeal carcinoma cells (NPC-039 and NPC-BM) [44]. Zhang et al., 2020 [47], reported cell cycle arrest in G2/M in A549 (human lung cancer) cells exposed to eriodictyol.

The cytostatic activity of these compounds has been described in the literature; however, their contribution to the cell cycle arrest of a complex matrix, such as *T. carnosus* extracts, and comparisons between different cell lines have been understudied. In this work, we report that the individual effect of each component and the phytochemical overall content do not allow for the prediction of the cytostatic effect of an extract or its role in antiproliferative activity. Further studies focused on the synergisms between compounds should be performed. In the particular case of the extracts under study, cell cycle arrest did not arise as a major contributor to the cell death process.

### 2.4. Evaluation of Extract- and Phytochemical-Induced Apoptosis/Necrosis in Caco-2 and HepG2 Cells

**Figure 5:**
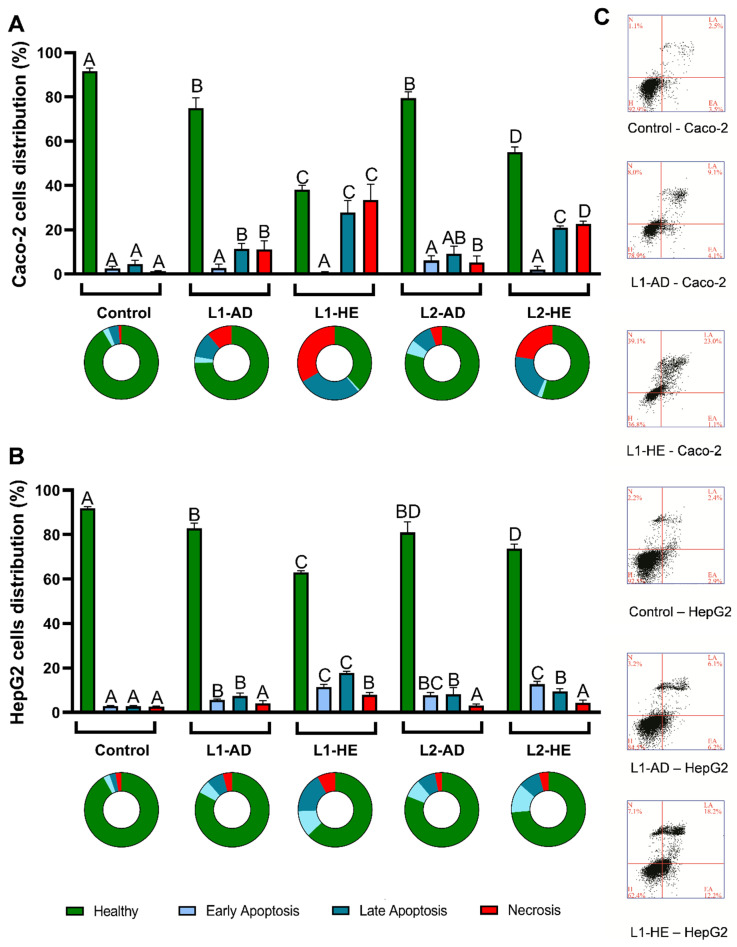
Assessment of apoptotic vs. necrotic cell death in Caco-2 (**A**) and HepG2 (**B**) cells exposed to *T. carnosus* extracts (100 µg/mL; extracts from 2020 harvest) for 24 h, evaluated through Annexin V-FITC/PI double staining. Significant statistical differences (when *p* < 0.05) between samples within each population are denoted with different letters (H: healthy; EA: early apoptosis; LA: late apoptosis; N: necrosis). Results are presented as mean ± SD and were obtained from three individual assays as those presented in panel (**C**).

In order to understand how the safety profile of *T. carnosus* extracts varies with location, extraction method, and cell line, the ability of the extracts to induce apoptosis and/or necrosis was studied in Caco-2 and HepG2 cells. The results are shown in Figure 5.

In Figure 5, it can be seen that within the various parameters under study (location, type of extraction, cell line), the extraction method induced the largest variability in apoptosis/necrosis induction. In Caco-2 cells (Figure 5A), the HE extracts induced a higher percentage of cells in late apoptosis (Annexin V-positive and PI-positive staining; Annexin^+^/PI^+^) and necrosis (Annexin-negative and PI-positive; Annexin^−^/PI^+^) than the AD extracts, while in HepG2 cells (Figure 5B), there was mainly an increase in the percentage of cells stained for early apoptosis (Annexin-positive and PI-negative; Annexin^+^/PI^−^) and late apoptosis. Regarding Caco-2 cells exposed to AD extracts, both extracts reduced the percentage of healthy cells from 93.01% (non-exposed cells) to ~75% and ~80% for L1-2020-AD and L2-2020-AD, respectively (Figure 5A).

However, the cell death induced by L1-2020-AD presented as a shift in the cell population toward late apoptosis (11.28%) and necrosis (11.05%), while exposure to L2-2020-AD increased the percentage of cells in early apoptosis (6.13%), late apoptosis (9.13%), and necrosis (5.32%). Thus, the reduction in the percentage of healthy cells was similar for the exposure to both extracts. 

It should be noted that despite the triggering of apoptosis and/or necrosis presented in Figure 5, at 100 µg/mL none of the extracts reduced the viability of Caco-2 cells exposed for 24 h (Figure 1), as assessed by an Alamar Blue assay. Regarding the HE extracts, a similar effect was observed for the extracts from different locations, with an increase in the percentage of cells undergoing late apoptosis and necrosis, this effect being greater in cells exposed to L1-2020-HE (Figure 5A), which is in line with the highest toxicity reported for this extract in Figure 1. In HepG2 cells, both the AD and HE extracts produced a similar pattern, with a significant increase in the percentage of cells undergoing early and late apoptosis, while only L1-2020-HE induced a significant increase in necrotic cells (Figure 5B). As observed in Caco-2 cells, the AD extracts induced a similar reduction in the percentage of healthy cells, resulting in an increase in the percentage of cells stained for early and late apoptosis. Likewise, in HepG2 cells, the L1-2020-HE extract also induced the highest cytotoxicity.

It should be highlighted that in HepG2 cells, both the AD and HE extracts produced less cytotoxicity than in Caco-2 cells (Figure 5). As shown in Figure 1, the exposure of Caco-2 cells to extracts at a concentration of 100 µg/mL for 24 h did not result in a reduction in cell viability. A similar conclusion was obtained for HepG2 cells exposed to the AD extracts, but the HE extracts produced significant toxicity at concentrations ≥75 µg/mL, resulting in lower IC_50_ values than in Caco-2 cells, as shown for L2-2020-HE, which had the lowest IC_50_ (92.25 µg/mL; 24 h; Figure 1J). This revealed that different results may arise depending on the cell viability method used, such as between assays dependent on the metabolic rate and the reducing potential of cells (e.g., Alamar Blue assay) and those that assess the presence/quantity of cell death biomarkers (e.g., by flow cytometry). Alamar Blue reagent allows for an estimation of cell viability based on its metabolism [54], which may provide an overestimation of cell viability. Caco-2 cells undergoing apoptosis that did not lose membrane integrity may still have been reducing Alamar Blue, while, in HepG2 cells, we saw a hyperpolarization of the mitochondrial membrane (Figure 3) that could have impaired energy production and limited the reducing potential, thus contributing to a decreased reduction of Alamar Blue. 

**Figure 6:**
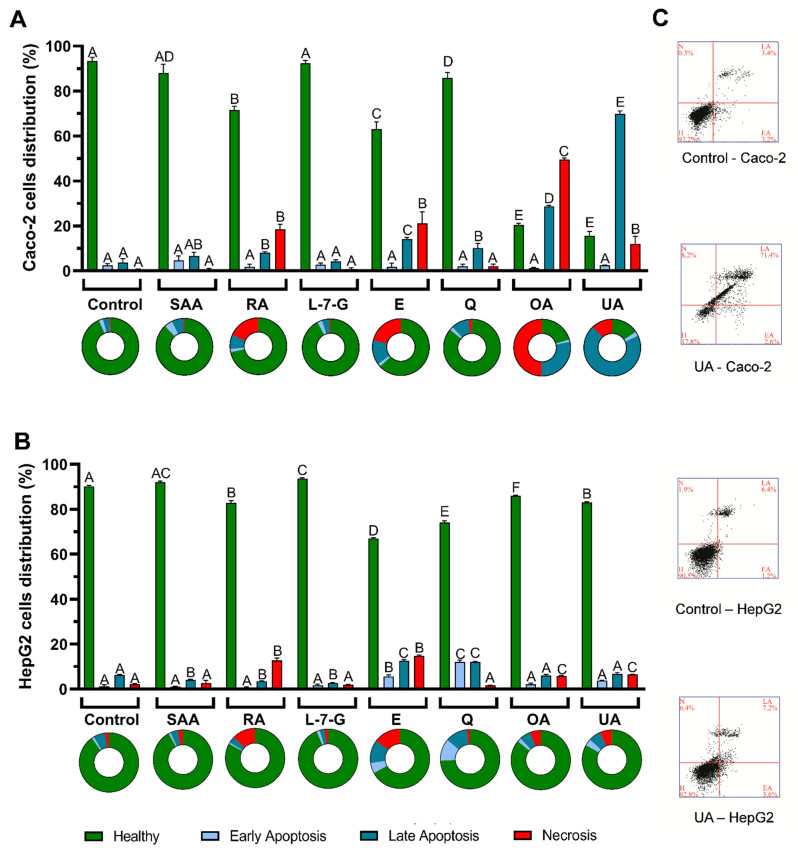
Assessment of apoptotic vs. necrotic cell death in Caco-2 (**A**) and HepG2 (**B**) cells exposed to *T. carnosus* extracts’ main components (at 50 µM for 24 h), evaluated through Annexin V-FITC/PI double staining. Significant statistical differences (when *p* < 0.05) between samples within the same population are denoted with different letters (H: healthy; EA: early apoptosis; LA: late apoptosis; N: necrosis). Results are presented as mean ± SD and were obtained from three individual assays as those presented in panel (**C**).

As discussed above, an increase in MMP can lead to mitochondrial swelling and rupture, with the further release of proapoptotic factors into the cytoplasm and triggering mitochondria-dependent apoptosis [41,42,43]. However, the formation of the apoptosome (a key step in the intrinsic apoptotic pathway) is highly dependent on ATP levels, and thus energy depletion leads to the interruption of the apoptosis signaling pathway, with the cells deviating into necrosis [51]. If ATP depletion was promoted by the hyperpolarization and subsequent mitochondrial membrane rupture, this would have contributed to the apoptosis and necrosis observed in the HepG2 cells exposed to L1-2020-HE extracts. Caco-2 cells exposed to the HE extracts presented no (L1 extracts) or low (L2 extracts) variation in the MMP, suggesting that another mechanism of action was underlying the higher necrotic death observed in these cells or that the time scale of the events was different from that occurring in HepG2 cells. Nevertheless, a study of Annexin/PI staining at different time points and for different concentrations is needed to clarify this difference. But, it is likely that the ROS increase was related to the cytotoxicity observed in both cell lines, while changes in the MMP variation may have explained the differences between cell lines. As an attempt to correlate the phytochemical composition with the differences observed between extracts, the effect of the main compounds on apoptosis and necrosis induction was also studied, and the results are presented in Figure 6.

UA was the compound that induced the largest increase in the percentage of cells undergoing late apoptosis (~70%) in Caco-2 cells (Figure 6). UA also induced necrosis (~12%) in Caco-2 cells. While UA-induced apoptosis was observed in Caco-2 cells (Figure 6A), in HepG2 cells, UA only slightly increased (*p* < 0.05) the percentage of necrotic cells (Figure 6B), which implied differences in the mechanisms of the UA-induced antiproliferative activity between cell lines (or the different time scale of the events) but is in line with the results obtained from the Alamar Blue assay in which UA induced higher toxicity in Caco-2 cells than in HepG2 cells (Figure 2). The mitochondria-dependent necrosis hypothesis described above was already reported by Lu et al., 2014 [51], for the action of 17.50 µM UA in DBTRG-05MG cells (human glioblastoma); however, it should be noted that the authors considered the events positively stained for PI and Annexin as necrotic cells.

In this study, necrotic events were considered as the cells that only stained positive for PI (corresponding to a loss of membrane integrity but no phosphatidylserine externalization), and double-stained events were considered as late apoptotic cells that presented phosphatidylserine externalization and the loss of membrane integrity, as also considered by other authors [55,56]. Nevertheless, it is known that programmed necrosis pathways such as necroptosis can also present double staining [55]. 

Although this was not addressed in the present study, future studies should address the signaling pathways associated with apoptosis and necrosis induction to better differentiate and understand the antiproliferative effect of *T. carnosus* extracts and their main phytochemicals. Regarding the OA effect, when compared to its isomer, UA, a similar reduction in healthy cells was observed in Caco-2 cells (Figure 6A); however, OA induced a higher percentage of cells in necrosis (~50%) than in late apoptosis (~29%) than UA. In HepG2 cells, OA induced a slight reduction in the percentage of healthy cells but to a lesser extent than in Caco-2 cells (Figure 6B), once again supporting the Alamar Blue results. While the pentacyclic triterpenoids were likely the main contributors to the cytotoxicity of the HE extracts, regarding the AD extracts, other compounds arose as the main effectors. Eriodictyol induced apoptosis and necrosis in Caco-2 and HepG2 cells, presenting the highest induction of cell death among the phenolic compounds tested (Figure 6). RA induced a significant increase in both late apoptosis and necrosis in Caco-2 cells, as well as necrosis in HepG2 cells. Correlating with the oxidative stress results, as seen in Figure 3, eriodictyol increased the intracellular ROS in HepG2 cells. However, while RA induced a loss in the MMP in HepG2 cells, eriodictyol produced mitochondrial membrane hyperpolarization in HepG2 cells. Furthermore, in Caco-2 cells, eriodictyol induced a loss of the MMP, and neither RA nor eriodictyol induced ROS increases. Thus, despite inducing similar effects on cell death (assessed by flow cytometry), these phytochemicals seem to induce different mechanisms.

Regarding quercetin, this flavonoid appeared to play a significant role in apoptosis induction. In Caco-2 cells, this effect was seen was a shift in the cell distribution into late apoptosis, while in HepG2 cells, 24 h of exposure produced a shift in the cell population to both early and late apoptosis, resulting in a large reduction in healthy cells (% of healthy Caco-2 cells: ~86%; HepG2 cells: ~76%). Quercetin did not induce variations in the MMP in Caco-2 or HepG2 cells but significantly increased the intracellular ROS in HepG2 cells, which was likely related to the increased cell death and may provide a justification for the different cytotoxicity patterns observed between the cell lines. L1-2020-HE, the extract with higher content of quercetin derivatives (Table 1), produced the highest increase in cells in late apoptosis of all the extracts tested. L-7-G did not induce cell death in either cell line. In fact, in HepG2 cells, a slight increase in the percentage of healthy cells was observed (Figure 6B). As mentioned above, the overall effect of the extracts was difficult to trace to a specific compound, so further studies are needed using combinations of phytochemicals to understand which synergisms occur and which can modulate changes in intracellular ROS and MMP and subsequently trigger the apoptotic and necrosis pathways. In a previous study [1] reporting the antiproliferative effect of a *T. carnosus* extract in Caco-2 cells, AD extracts induced a significant increase in cells in late apoptosis; however, this effect was only observed at higher concentrations (400, 500, and 600 µg/mL) than the one here used (100 µg/mL). To best of our knowledge, this is the first study reporting the effect of the type of extraction, harvest location, and main components of *Thymus* spp. extracts on oxidative stress, MMP modulation, and apoptosis/necrosis induction evaluated in two cell lines. Other authors have addressed the mechanisms of the antiproliferative activity of *Thymus* spp. extracts in various cell line models using fewer variables. Using a hydroalcoholic extract of *T. vulgaris* and human non-small-cell lung carcinoma cells (H460), Oliviero et al., 2016 [57], reported a reduction in cell viability, with no effect on cell cycle progression, but with necrosis induction (only PI-positive staining). Also using *T. vulgaris*, Al-Menhali et al., 2015 [58], report increased activities of caspase-3 and caspase-7 in HCT-116 cells exposed to an aqueous extract. However, the effect was observed at higher concentrations (400 and 600 µg/mL) than the ones here used (100 µg/mL) [58]. In addition to being the most consumed thyme species, *T. vulgaris* is also the most frequently studied thyme species, and its antiproliferative effect was also evaluated in a breast cancer cell line (T-47D). The *T. vulgaris* ethanolic extract was effective in inducing apoptosis at low concentrations (10 and 20 µg/mL) [59]. Also in breast cancer models but using a methanolic extract of *Thymus serpyllum*, Bozkurt et al., 2012 [60], reported that cell exposure to the extract for 24 h significantly reduced cell viability at concentrations ≥50 µg/mL in MCF-7 cells and at concentrations ≥250 µg/mL in MDA-MB-231 cells. The antiproliferative activity was dependent on DNA fragmentation and increases in caspase-3 and caspase-7 activation in MDA-MB-231 cells [60]. 

Two final remarks should be provided regarding the different responses of Caco-2 and HepG2 cells to the *T. carnosus* extracts. In this manuscript, we described the increased cytotoxicity induced by the extracts in the hepatocyte model using the Alamar Blue method but not in the flow cytometry studies. Considering that the species under study is a herb, and its potential use as a functional food implies oral administration, the gastrointestinal tract is the primary point of contact between xenobiotics and the human organism, serving as the initial semipermeable barrier that regulates nutrient and xenobiotic uptake. Then, once in the systemic circulation, the first passage effect occurs in the liver, where hepatocytes act as cells specialized in xenobiotic metabolism [61]. Thus, while the intestinal tract is exposed to the full dietary content, hepatocytes are only exposed to the absorbed components. This was transposed to in vitro experimental models where, using transwell insert-based methodologies, Caco-2 cells, a well-established cell line model of the intestinal epithelium, and HepG2 cells, which simulate hepatocyte metabolism, were used [62]. In this coculture model, Caco-2 cells are seeded in the insert and are allowed to differentiate into enterocytes, expressing an apical membrane, facing the upper compartment, and a basolateral membrane, facing the lower compartment. The lower compartment comprises the well in the companion plates, where the transwell inserts are placed, which contains HepG2 cells at the bottom. In this model, only Caco-2 cells are exposed to the initial crude sample, while HepG2 cells are only exposed to compounds that permeate the Caco-2 monolayer [62], likely providing more accurate results. Due to the scarce knowledge regarding the effect of *T. carnosus* extracts in both the intestinal epithelium and hepatic tissue, the present study aimed to establish a baseline for the safety profile for using this species in the human diet. Due to the higher resistance of Caco-2 cells to the extracts’ activity, they should be assessed in future studies using refined in vitro models that intend to mimic in vivo metabolism and whether the Caco-2 monolayer can modulate the absorption of phytochemicals, thus reducing/modifying the exposure of HepG2 cells to these products.

A second note is related to the role of hepatic metabolism. Hepatocytes are cells specialized in the biotransformation of xenobiotics, being equipped with enzymatic tools such as phase I and phase II enzymes that regulate the detoxification of both endogenous and exogenous molecules [63]. However, this metabolic process may culminate in the production of reactive byproducts, which contribute to the cytotoxicity observed for phytochemicals [61]. This highlights the scarce knowledge of this topic and the need for additional studies focused on different time points and concentrations, addressing the molecular pathways underlying this effect. For these reasons, further studies are needed in order to assess the potential of *T. carnosus* as a new source of nutraceuticals, namely, studies of intestinal permeability, bioavailability, and bioaccumulation through the intestinal barrier, as well as hepatic metabolization, in order to better assess the phytochemicals (or their metabolites) that could potentially exert hepatotoxicity. Following the initial assessment presented in this paper, more refined models (such as the transwell insert method) should be applied.

Although further evaluation of *T. carnosus* bioactivities using different experimental models is needed, based on the results we obtained for *T. carnosus* bioactivities, it is clear that this species has the potential for use in both the food and pharmaceutical industries, particularly as a source of nutraceuticals. Given the near-threatened status of *T. carnosus*, we expect that by increasing its value in various industries, it will raise awareness of the need for its conservation, encourage additional studies on its use as a crop, promote a sustainable cultivation approach, and promote its application for the benefit of human health.

## 3. Materials and Methods

### 3.1. Materials

Rosmarinic acid, salvianolic acid A, quercetin, and ursolic acid were purchased from Sigma-Aldrich/Merck (Algés, Portugal); luteolin-7-glucoside was obtained from Extrasynthese^®^ (Genay, France); and eriodictyol and oleanolic acid were obtained from Santa Cruz Biotechnology Inc. (Frilabo, Porto, Portugal). Dulbecco’s modified Eagle medium (DMEM), versene, trypsin-EDTA, penicillin, streptomycin, fetal bovine serum (FBS), L-glutamine, and sodium pyruvate were obtained from Gibco (Alfagene, Lisboa, Portugal). Alamar Blue^®^ was purchased from Invitrogen, Life-Technologies (Alfagene, Lisboa, Portugal). Dichlorodihydrofluorescein diacetate (DCFDA), 5,5″,6,6″-tetrachloro-1,1″,3,3″-tetraethylbenzimidazolylcarbocyanineiodide (JC-1), and Annexin-V-FITC were purchased from Thermo Fisher Scientific (Alfagene, Lisboa, Portugal). RNAse was obtained from NZYtech (Lisboa, Portugal). Flow cytometry solutions and consumables were obtained from BD—Becton Dickinson (Enzifarma, Lisboa, Portugal). Other salts and reagents not mentioned above were obtained from Sigma-Aldrich/Merck (Algés, Portugal).

### 3.2. Plant Material, Sample Preparation and Stock Solutions

In the present study, we used *T. carnosus* extracts obtained from plant material (aerial parts: leaves and stems) harvested in 2018, 2019, and 2020, from Arrábida Natural Park (L1) and the UTAD’s botanical garden (L2), which were previously prepared and characterized by Martins-Gomes et al., 2023 [3]. The harvests performed at L1 were authorized (license nos. 867/2018/RECOLHA; 868/2018/RECOLHA; 723/2019/RECOLHA; 723/2019/RECOLHA; 198/2020/RECOLHA; 199/2020/RECOLHA) and supervised by the Portuguese Institute for Nature Conservation and Forests (ICNF). The plant material harvested at L1 and L2 was authenticated by the UTAD’s Botanical Garden office (Vila Real, Portugal), originating from voucher specimen no. HVR22496 (L1) and no. HVR21093 (L2).

After harvest, the plant material was cleaned of any debris, rinsed with distilled water, weighed, and frozen for further lyophilization (Dura Dry TM P freeze-drier; −45 °C; 250 mTorr). Lyophilized material was then ground and used to obtain AD and HE extracts, which were prepared as described by Martins-Gomes et al., 2023 [3]. Briefly, for AD extracts, 150 mL of distilled water was added to 0.5 g of lyophilized plant material, and this mixture was then heated to 100 °C under agitation and held at this temperature for 20 min. The mixture was then removed from the heat and allowed to cool down to room temperature, followed by two filtration steps, first using a Whatman no. 4 filter and then with a fiberglass filter (1.2 μm; obtained from VWR International Ltd., Alfragide, Portugal). For HE extracts, an exhaustive extraction method was used, where 50 mL of a hydroethanolic solution (80% ethanol: 20% water; % *v/v*) was added to 0.5 g of lyophilized plant material. The mixture was agitated on an orbital shaker (150 rpm) for one hour at room temperature, followed by centrifugation (7000 rpm, Sigma Centrifuges 3–30 K, St. Louis, MO, USA). After this, the supernatant was collected, and the pellet was used to repeat the extraction procedure for a total of three extractions. The three supernatants were combined and filtered as described above for AD extracts. Both extracts were concentrated to 100 mL using a rotary evaporator (35 °C), which also allowed the removal of ethanol from HE extracts. All extracts were lyophilized, weighed, and stored until further use. Three extractions were performed for each sample and each extraction method.

The phytochemical profile of all extracts was assessed using HPLC-DAD and HPLC-ESI-MS^n^. The equipment and chromatographic conditions were described by Martins-Gomes et al., 2023 [3]. Identification of phenolic compounds was based on retention time, UV–VIS spectra, fragmentation pattern, comparison to commercial standards (when available), or comparison to the literature. Pentacyclic triterpenoids oleanolic (OA) and ursolic acid (UA) were identified by comparison of retention time and UV–VIS spectra with commercial standards. The quantification was performed by HPLC-DAD, using the calibration curves of commercial standards. When the exact commercial standard was not available, the calibration curve of the compound with the highest structural similarity was used. For example, caffeic acid was quantified using a caffeic acid calibration curve. For the different glycoside derivatives, luteolin derivatives were quantified as luteolin-7-*O*-glucoside, quercetin derivatives were quantified as quercetin-3-*O*-glucoside, and eriodictyol derivatives were quantified as eriodictyol-7-*O*-glucoside. Phenolic acids (RA and salvianolic acids) were quantified using an RA calibration curve [3].

Stock solutions (10 mg/mL) of AD and HE extracts were prepared in PBS and 10% DMSO (in PBS), respectively. The extracts dissolved completely under the above conditions, and no precipitation was observed in stock solutions or on further dilution in culture media. With the goal of studying the contribution of the major compounds to the bioactivities observed for the total extracts, various phytochemicals were also analyzed. The selected compounds were salvianolic acid A (SAA), rosmarinic acid, luteolin-7-*O*-glucoside (L-7-G), eriodictyol (E), quercetin (Q), oleanolic acid, and ursolic acid. Stock solutions were prepared in DMSO at 10 mM. The final concentration of DMSO in the test solutions did not exceed 1%, which was previously shown to have no effect on cell viability. 

### 3.3. Cell Culture Maintenance and Cell Viability Assessment

The safety profile of *T. carnosus* extracts was assessed in Caco-2 and HepG2 cells. Caco-2 (human colon adenocarcinoma cell line) were obtained from CLS (Cell Lines Service, Eppelheim, Germany), and HepG2 (human hepatocellular carcinoma cell line) were obtained from ATCC (American Type Culture Collection, Manassas, VA, USA).

Cell culture maintenance, handling, subculturing, and seeding were performed as described by Silva et al., 2020 [5], and cell viability was assessed using an Alamar Blue^®^ assay, as described by Andreani et al., 2014 [64]. Concentrations ranged between 100 and 1000 µg/mL for AD extracts in both cell lines and 100 to 300 µg/mL or 50 to 150 µg/mL for HE extracts in Caco-2 and HepG2 cells, respectively. The effect of individual compounds on cell viability was assessed for concentrations ranging from 50 to 300 µM. All test solutions were prepared by diluting the samples in FBS-free culture media from the stock solutions described in Section 3.2, just prior to being used.

### 3.4. Flow Cytometry Assessment of Intracellular ROS, Mitochondrial Membrane Potential, Cell Cycle, and Cell Death

Flow cytometry was used to analyze the effect of *T. carnosus* extracts on intracellular ROS, modulation of mitochondrial membrane potential (MMP), cell cycle arrest, and apoptotic/necrotic cell death induction.

Both one- and two-color assays were performed using a BD Accuri™ C6 cytometer (Becton Dickinson, Franklin Lakes, NJ, USA), and 10,000 gated events were collected from each sample [1]. For flow cytometry assays, cells were handled as described previously [1] but seeded at a density 5 × 10^4^ cells/mL (750 μL/well) in 12-well plates. Caco-2 and HepG2 cells were incubated for 24 h with the extracts (100 µg/mL) or individual phytochemicals (50 µM) diluted in FBS-free culture media h. After incubation, test solutions were removed; the cells were washed with PBS and detached from the plates using trypsin-EDTA. Once transferred to centrifuge tubes, the cells were centrifuged (bench micro-centrifuge; 3000 rpm; 3 min), the supernatant was discarded, and a new wash cycle was performed. The cells were then equally divided for each assay. A negative control was used in all assays consisting of nonexposed cells incubated only with culture media. 

Intracellular ROS level assessment was performed as described by Silva et al., 2022 [65], using dichlorodihydrofluorescein diacetate (DCFDA). Cells obtained in the previous step were centrifuged, the supernatant discarded, and 200 µL of DCFDA (10 µM in FBS-free DMEM) was added. Cells were incubated for 45 min (37 °C, in the dark), washed to remove excess probe, and resuspended in PBS for data acquisition.

Mitochondrial membrane potential (MMP) was evaluated using 5,5″,6,6″-tetrachloro-1,1″,3,3″-tetraethylbenzimidazolylcarbocyanineiodide (JC-1). Cells were processed as described for DCFDA but incubated with 200 µL of JC-1 (2 µM, in FBS-free DMEM) for 20 min (37 °C, in the dark) [65]. As JC-1 is a ratiometric indicator of MMP variations, the results are presented as the ratio of mean FL2/FL1 fluorescence intensities, normalized by the negative control (nonexposed cells). Positive control was performed using FCCP (5 µM; carbonyl cyanide *p*-trifluoromethoxyphenylhydrazone), which induced a significant reduction in MMP in Caco-2 cells (0.06 ± 0.01; reduction in MMP of ~94%) and HepG2 cells (0.10 ± 0.01; reduction in MMP of ~90%).

For the assessment of apoptotic/necrotic cell death, an Annexin V-FITC (Thermo Fisher Scientific, Waltham, MA, USA) and propidium iodide (PI; Merck, Darmstadt, Germany) double-staining assay was used to evaluate the apoptotic/necrotic cell death induced by *T. carnosus* extracts and by individual phytochemicals, as described previously [1]. Briefly, after detachment and washing, the supernatant was discarded, and 200 µL of Annexin V-FITC (1:200 dilution in Annexin-binding buffer [10 mM HEPES sodium salt, 150 mM NaCl, 5 mM KCl, 5 mM MgCl_2_, and 1.8 mM CaCl_2_; pH = 7.4]) was added. Cells were incubated for 20 min (room temperature, in the dark). After incubation, 5 µL of propidium iodide (PI; 50 µg/mL) was added, the cells were incubated for 5 min on ice (in the dark), and the data were acquired.

Cell cycle arrest assessment was performed as described in [1]. Cells were processed as described above until cell detachment and centrifuged, after which the second washing step was performed with ice-cold PBS (0 °C). After centrifugation, the supernatant was discarded, and cells were fixed with 500 µL of ethanol:PBS solution (70:30, % *v/v*, previously cooled at −20 °C) for at least 4 h at −20 °C. After fixation, two washing cycles were performed: the first with ice-cold PBS (0 °C) and the second with room-temperature PBS, followed by the addition of 200 µL of a PBS solution containing PI (50 µg/mL), RNAse A (50 µg/mL), and Triton X-100 (0.1%). Cells were incubated for 30 min at 37 °C, and then cells were washed once with PBS to remove the staining solution. After resuspension in PBS, the data were acquired.

### 3.5. Data and Statistical Analysis

The results are presented as mean ± SD of at least three independent assays. The calculation of IC_50_ values was performed as described by Silva et al., 2020 [5]. Data analysis, graphical design, and statistical analysis were performed using Microsoft Office Excel (Professional Plus 2019; Microsoft Corporation, Washington, DC, USA) and GraphPad Prism (Version 8; GraphPad Software Inc., San Diego, CA, USA). Flow cytometry data analysis was performed using BD Accuri™ C6 Software, version 1.0.264.21 (Becton Dickinson, San Diego, CA, USA).

## 4. Conclusions

In the present work, the effects of the extraction method, geographical location, and interannual variability on the antiproliferative activity of *T. carnosus* extracts were assessed for the first time. The HE extracts were more cytotoxic to Caco-2 and HepG2 cell lines than the AD extracts. However, it was found that apoptosis and necrosis were induced at concentrations considered noncytotoxic in an Alamar Blue assay, reinforcing the need to deepen the studies of natural products, which have been superficial studies on their bioactivities. It was proven that the cell death induced by the extracts was dependent on the increase in intracellular ROS levels and the modulation of MMP and on the cell line used. The hepatocyte model used (HepG2 cells) showed higher sensitivity to the extract-induced antiproliferative activity in the Alamar Blue assay but not in the flow cytometry assays. It was found that the bioactivities were highly dependent on the overall phytochemical composition rather on the effect of a single component. However, additional studies on *T. carnosus* extracts’ phytochemical composition, including the identification of additional phytochemicals and their content in other water-soluble constituents (e.g., sugars, proteins), as well as further studies on potential bioactivities, may provide the opportunity to find additional correlations between phytochemical composition and bioactivities. To the best of our knowledge, this study addressed, for the first time, an association between phytochemical composition and the cellular modulation of oxidative stress, MMP, and apoptosis/necrosis induced by thyme extracts. These results highlight the need to improve the methodologies used to evaluate the safety profile of natural compounds and to understand how geographical and climatic changes may impact their bioactivities, which is mandatory for products to be included in the human diet. 

## Figures and Tables

**Table 1:** Main components quantified in *T. carnosus* AD and HE extracts obtained from plant material harvested in 2020 (from data reported in [3]).

Component	Concentration (µM) Present in 100 µg/mL of Extract			
	L1-2020-AD	L1-2020-HE	L2-2020-AD	L2-2020-HE
**Phenolic acids**				
Rosmarinic acid	3.04	7.39	2.24	6.15
Salvianolic acid A isomer	2.14	3.62	1.52	2.59
Salvianolic acid K	1.85	2.23	1.20	1.63
Salvianolic acid B/E isomer	0.19	0.48	0.19	0.32
**Flavonoid derivatives**				
Luteolin-*O*-hexoside isomer	3.08	4.38	1.93	3.63
Quercetin-*O*-hexoside	2.46	5.59	0.95	3.27
Quercetin-*O*-hexoside-hexuronide	0.35	0.40	0.25	0.40
Eriodictyol-*O*-hexoside isomer	0.49	0.35	0.27	0.28
Eriodictyol-*O*-hexoside isomer	0.50	0.53	0.30	0.32
Luteolin-*O*-hexoside-hexoside isomer	0.47	0.35	0.24	0.21
Luteolin-*O*-hexoside isomer	0.33	0.20	0.17	0.14
Acetyl-luteolin-*O*-hexoside-pentoside	0.27	0.34	0.12	0.17
Luteolin-*O*-hexoside-hexoside isomer	0.22	0.28	0.17	0.30
Luteolin-*O*-hexoside-*O*-pentoside	0.26	0.20	0.17	0.19
**Pentacyclic triterpenoids**				
Oleanolic acid	0	12.22	0	9.04
Ursolic acid	0	11.35	0	8.87
Total salvianolic acids	4.19	6.34	2.91	4.55
Total eriodictyol derivatives	0.99	0.89	0.57	0.60
Total luteolin derivatives	4.63	5.74	2.80	4.65
Total quercetin derivatives	2.82	5.99	1.20	3.67
Total triterpenoids (OA + UA)	0	23.57	0	17.91

Notes: OA: oleanolic acid; UA: ursolic acid. Identification and quantification of phytochemicals’ were performed using HPLC-DAD-ESI-MS^n^, as described in [3], based on retention time, MS fragmentation pattern, UV–VIS spectra, and comparison to commercial standard and/or the literature. In first column, different classes of compounds are denoted by different colors, as follows: phenolic acids (blue), flavonoid derivatives (green) and pentacyclic triterpenoids (orange).

## Data Availability

Data are contained within the article.
